# A Novel Prognostic Score Based on Artificial Intelligence in Hepatocellular Carcinoma: A Long-Term Follow-Up Analysis

**DOI:** 10.3389/fonc.2022.817853

**Published:** 2022-05-31

**Authors:** Xiaoli Liu, Xinhui Wang, Lihua Yu, Yixin Hou, Yuyong Jiang, Xianbo Wang, Junyan Han, Zhiyun Yang

**Affiliations:** ^1^Center for Integrative Medicine, Beijing Ditan Hospital, Capital Medical University, Beijing, China; ^2^Institute of Infectious Diseases, Beijing Ditan Hospital, Capital Medical University, Beijing, China

**Keywords:** liver cancer, artificial neural networks, peripheral CD8 T cells, prognosis system, immune score

## Abstract

**Objective:**

T cell immunity plays an important role in anti-tumor effects and immunosuppression often leads to the development and relapse of cancer. This study aimed to investigate the effect of T cell numbers on the long-term prognosis of patients with hepatocellular carcinoma (HCC) and construct an artificial neural network (ANN) model to evaluate its prognostic value.

**Methods:**

We enrolled 3,427 patients with HCC at Beijing Ditan Hospital, Capital Medical University, and randomly divided them into two groups of 1,861 and 809 patients as the training and validation sets, respectively. Cox regression analysis was used to screen for independent risk factors of survival in patients with HCC. These factors were used to build an ANN model using Python. Concordance index, calibration curve, and decision curve analysis were used to evaluate the model performance.

**Results:**

The 1-year, 3-year, 5-year, and 10-year cumulative overall survival (OS) rates were 66.9%, 45.7%, 34.9%, and 22.6%, respectively. Cox multivariate regression analysis showed that age, white blood cell count, creatinine, total bilirubin, γ-GGT, LDH, tumor size ≥ 5 cm, tumor number ≥ 2, portal vein tumor thrombus, and AFP ≥ 400 ng/ml were independent risk factors for long-term survival in HCC. Antiviral therapy, albumin, T cell, and CD8 T cell counts were independent protective factors. An ANN model was developed for long-term survival. The areas under the receiver operating characteristic (ROC) curve of 1-year, 3-year, and 5-year OS rates by ANNs were 0.838, 0.833, and 0.843, respectively, which were higher than those of the Barcelona Clinic Liver Cancer (BCLC), tumor node metastasis (TNM), Okuda, Chinese University Prognostic Index (CUPI), Cancer of the Liver Italian Program (CLIP), Japan Integrated Staging (JIS), and albumin–bilirubin (ALBI) models (P < 0.0001). According to the ANN model scores, all patients were divided into high-, middle-, and low-risk groups. Compared with low-risk patients, the hazard ratios of 5-year OS of the high-risk group were 8.11 (95% CI: 7.0-9.4) and 6.13 (95% CI: 4.28-8.79) (P<0.0001) in the training and validation sets, respectively.

**Conclusion:**

High levels of circulating T cells and CD8 + T cells in peripheral blood may benefit the long-term survival of patients with HCC. The ANN model has a good individual prediction performance, which can be used to assess the prognosis of HCC and lay the foundation for the implementation of precision treatment in the future.

## Introduction

Primary liver cancer was the sixth most common cancer and the third leading cause of cancer-related deaths worldwide in 2020, with an estimated 906,000 new cases and 830,000 cancer-related deaths (1). The 5-year net survival was in the 10%–19% range in most areas around the world (2). With an aging of and increase in the world’s population, deaths due to liver cancer are increasing. It is predicted that the number of liver cancer deaths will reach 1,679,630 by 2040, an increase of 85.4% over 2020 (3). Hepatocellular carcinoma (HCC) accounts for 75%–85% of all primary liver cancers (4).

Currently, the commonly used treatments for HCC include surgical resection, liver transplantation, local ablation therapy (radiofrequency ablation, microwave ablation, cryoablation, percutaneous anhydrous alcohol injection), transarterial chemoembolization (TACE), and targeted therapy (5). Curative therapy should be selected as much as possible for early HCC, such as liver resection, liver transplantation, or ablation; the 5-year overall survival (OS) rate of HCC patients receiving curative therapy can reach 60%–70% (6, 7). However, because liver cancer is mostly diagnosed in the intermediate and advanced stages, only a few patients can choose curative therapy. In a multicenter cohort study of 8,656 patients, only 10% of newly diagnosed HCC patients were recommended for resection (8). The availability of liver transplantations is also limited by the lack of donors. Therefore, most HCC patients can only receive local treatment, such as TACE or palliative treatment and their 5-year OS is reduced by more than half to less than 30% (9). The high mortality of HCC patients remains a key clinical problem; therefore, the identification of prognostic indicators and model construction are used to predict the outcome.

Early intervention based on prediction systems and risk stratification is an effective strategy for improving the survival of HCC patients. At present, the staging systems for predicting and evaluating the prognosis of HCC patients include the tumor node metastasis (TNM) stage (10), Barcelona Clinic Liver Cancer (BCLC) stage (11), Okuda grade (12), Cancer of the Liver Italian Program (CLIP) score (13), Chinese University Prognostic Index (CUPI) (14), Japan Integrated Staging (JIS) (15), and albumin–bilirubin (ALBI) grade (16). The predictors of these prognostic models mainly focus on tumor burden, liver function, performance status, and so on. However, these factors mainly focus on the differences between the characteristics of tumors and cannot explain the interaction between the tumor and host immune response. Previous studies have reported that high densities of CD3 and CD8 immune cells in immunohistochemical sections of colorectal cancer (CRC) patients improve disease-free survival (DFS) and OS rates (17). Moreover, the type, density, and location of immune cells in CRC had a superior prognostic value and were independent of the TNM stage. Budhu et al. (18) revealed that the biological behavior of liver cancer is related to the unique immune response characteristics of the liver microenvironment, indicating that immune cells and immune responses may be related to the prognosis of patients with liver cancer. However, the current results on the relationship between outcomes and immune cells are inconsistent. Gabrielson et al. (19) demonstrated that the density of tumor-infiltrating CD3 and CD8 T cells could predict the recurrence of HCC in patients who underwent a hepatectomy (CD3, odds ratios (OR) = 5.8; CD8, OR= 3.9), and was independent of other predictive clinicopathological factors, such as vascular invasion and HCC cell differentiation. However, some studies have shown that tumor-infiltrating CD3, CD4, and CD8 T cells in HCC patients were not related to OS and DFS after resection, whereas high-density cytotoxic CD8 T cells (CTL) and low-density regulatory T cells (Tregs) were independent prognostic factors for improving OS and DFS (20). Most of these studies on immune cells and the prognosis of liver cancer are on patients after hepatectomy or liver transplantation; however, the relationship between immune cells and prognosis in unresectable patients is not clear.

Artificial neural networks (ANNs), as a form of machine learning, have been used for the prognostic evaluation of various tumors and have a great application prospect (21–23). Using machine learning to construct a prognostic system and stratify the risk of long-term survival of HCC patients is an effective strategy to implement precision therapy. This study aims to analyze the relationship between T cells and the prognosis of HCC and establish a prediction model for the long-term survival of HCC patients with immune indexes using ANNs, which can accurately identify populations at a high risk of death and carry out an early intervention to reduce patient mortality.

## Materials and Methods

### Patients

A total of 3,427 patients with first-diagnosed primary liver cancer who were hospitalized in Beijing Ditan Hospital, Capital Medical University, between January 2008 and June 2017 were enrolled retrospectively. This study was approved by the Ethics Committee of Ditan Hospital. The inclusion criteria were as follows: (1) patients diagnosed with primary liver cancer with or without chronic liver diseases and (2) their ages were between 18–75 years. We excluded patients with (1) cholangiocarcinoma (n = 213), (2) metastatic liver cancer (n = 96), (3) other types of tumors (n = 67), (4) lost to follow-up (n = 201), and (5) incomplete clinical data (n = 180). Finally, 2,670 patients were randomly divided into a training set (n = 1,861) and a validation set (n = 809). The diagnostic criteria for HCC are in accordance with the criteria of the Asia-Pacific clinical guidelines for HCC (24).

### Clinical and Laboratory Parameters

We recorded the clinical information including the gender, age, family history of HCC, history of smoking and alcohol abuse, liver cirrhosis status, medical comorbidities (diabetes, hypertension, hyperlipidemia and coronary artery disease), and aetiology of HCC (HBV, HCV, alcohol abuse and others). We also obtained blood test results from the clinical laboratory including routine blood examination, liver function, serum lipid level, serum alpha fetoprotein (AFP) levels, c-reactive protein, creatinine, prothrombin activity, and international standardized ratio levels. The peripheral blood was sucked and stained with MULTITEST CD45-Percp/CD3-FITC/CD4-APC/CD8-PE TruCount four-color kit (BD Biosciences) in clinical laboratory. We extracted the T cell, CD4 T cell, and CD8 T cell counts before the treatment. Tumor factors included tumor number, maximum tumor size, vascular invasion, and tumor metastasis based on the imaging data at enrollment.

### Follow-Up and Endpoint

The CT or MRI scan, ultrasonography, or serum AFP tests were performed every 3 months. The definition of progression conformed with the mRECIST criteria (25). The occurrence of vascular metastasis or extrahepatic diffusion was also considered as progression. Survival time was defined as the time from admission to death or final follow-up on December 31, 2019.

### Statistical Analysis

Statistical analysis was performed using IBM SPSS Statistics for Windows version 21.0. T test or Mann–Whitney U test was used for quantitative data comparison. Fisher’s exact or χ^2^ inspection was used for qualitative data comparison. Cox univariate and multivariate analyses (forward, maximum likelihood ratio) were used to screen the risk factors of death in patients with liver cancer. The ANN model was created using Python. Finally, the ANN model was compared with existing routine prognosis systems: TNM stage (10), BCLC stage (11), Okuda grade (12), CLIP score (13), CUPI (14), JIS (15), and ALBI grade (16). C-index and the areas under receiver operating characteristic (ROC) curve (AUC) and time-dependent ROC curve were used to test the discrimination of the models. To test the calibration degree of the model, the Hosmer–Lemeshow test was applied and a calibration curve was drawn. Decision curve analysis (DCA) was used to compare the clinical net benefit and performance improvement of this model with those of the above models. R version 3.3.2 was used for data analysis, and rms, survival, survminer, rmda, pROC, ggplot2, and timeROC packages were used. All tests were considered to be statistically significant at p < 0.05.

## Results

### Patient Characteristics

We enrolled 2,670 patients between 2008 and 2017 and randomly divided them into training (n = 1,861) and validation (n = 809) groups. Among them, 2,249 (84.2%) were infected with hepatitis B virus (HBV), 15 of them were coinfected with hepatitis C virus (HCV), and 160 had complications from chronic alcohol consumption (**Table 1**). In addition, 242 patients (9.1%) were infected with hepatitis C, 25 of whom had complications from chronic alcohol consumption. 282 patients (10.6%) had liver cancer caused by alcoholic liver disease, and 97 (3.6%) had other causes, including 21 with autoimmune liver disease, 11 with nonalcoholic fatty liver disease, and 64 with occult liver cancer. Among HCC patients with HBV, 1,920 (71.9%) received antiviral therapy, 1,250 (46.8%) achieved HBV DNA < 500 IU/mL, and 1,466 (54.9%) achieved HBeAg seroconversion. There were no significant differences in the demographic characteristics, past history, laboratory indicators, tumor characteristics, and treatment methods between the training and validation groups.

**Table 1:** Baseline demographics and clinical characteristics of patients with hepatocellular carcinoma.

	Total (n = 2670)%/Median (IQR)	Training cohort (n = 1861)%/Median (IQR)	Validation cohort (n = 809) %/Median (IQR)	*P* values
**Patients background**				
Age, yr (mean ± SD)	55.67 ± 10.49	56.79 ± 10.58	56.39 ± 10.28	0.364
Gender (male/female)	2079/591 (77.9%/22.1%)	1454/407 (78.1%/21.9%)	625/184 (77.3%/22.7%)	0.617
Family history of HCC (Yes/No)	91/2579 (3.4%/96.6%)	63/1798 (3.4%/96.6%)	28/781 (3.5%/96.5%)	0.921
Smoking history (Yes/No)	1102/1568 (41.3%/58.7%)	774/1087 (41.6%/58.4%)	328/481 (40.5%/59.5%)	0.614
Alcohol abuse (Yes/No)	1050/1620 (39.3%/60.7%)	744/1117 (40%/60%)	306/503 (37.8%/62.2%)	0.295
Liver cirrhosis (Compensated/Decompensated/Non)	2123/341/206 (79.5%/12.8%/7.7%)	1472/239/150 (79.1%/12.8%/8.1%)	651/102/56 (80.5%/12.6%/6.9%)	0.576
Diabetes(Yes/No)	578/2092 (21.6%/78.4%)	404/1457 (21.7%/78.3%)	174/635 (21.5%/78.5%)	0.908
Hypertension(Yes/No)	686/1984 (25.7%/74.3%)	497/1364 (26.7%/73.3%)	189/620 (23.4%/76.6%)	0.069
Hyperlipidemia(Yes/No)	194/2476 (7.3%/92.7%)	133/1728 (7.1%/92.9%)	61/748 (7.5%/92.5%)	0.719
Coronary artery disease(Yes/No)	77/2593 (2.9%/97.1%)	56/1891 (3.0%/97.0%)	21/788 (2.6%/97.4%)	0.558
**Aetiology**				
HBV (Yes/No)	2249/421 (84.2%/15.8%)	1560/301 (83.8%/16.2%)	689/120 (85.2%/14.8%)	0.382
HCV (Yes/No)	242/2428 (9.1%/90.9%)	180/1681 (9.7%/90.3%)	62/747 (7.7%/92.3%)	0.097
Alcohol abuse (Yes/No)	282/2388 (10.6%/89.4%)	201/1660 (10.8%/89.2%)	81/728 (10.0%/90.0%)	0.542
Others (Yes/No)	97/2573 (3.6%/96.4%)	68/1793 (3.7%/96.3%)	29/780 (3.6%/96.4%)	0.93
**HBV-related indicators at baseline**				
HBeAg (Positive/Nagitve/NA)	783/1466/421 (29.3%/54.9%/15.8%)	540/1020/301 (29.0%/54.8%/16.2%)	243/446/120 (30.0%/55.1%/14.8%)	0.653
HBV-DNA, IU/ml (≥ 500/<500/NA)	999/1250/421 (37.4%/46.8%/15.8%)	681/879/301 (36.6%/47.2%/16.2%)	318/371/120 (39.3%/45.9%/14.8%)	0.371
Antiviral therapy (Yes/No/NA)	1920/329/412 (71.9%/12.3%/15.8%)	1326/234/301 (71.3%/12.6%/16.2%)	594/95/120 (73.4%/11.7%/14.8%)	0.515
**Laboratory parameters**				
White blood cells (10^9^/L)	4.92 ± 2.59	4.91 ± 2.60	4.94 ± 2.55	0.762
NLR	2.44 (1.62, 4.0)	2.45 (1.60, 4.03)	2.42 (1.65, 3.96)	0.856
Hemoglobin (g/L)	123.72 ± 25.24	123.68 ± 25.28	123.82 ± 25.18	0.895
Platelets (10^9^/L)	92.4 (59.0, 143.43)	91.1 (58.65, 144.0)	94.0 (60.15, 142.0)	0.457
ALT (U/L)	33.0 (22.2, 54.1)	33.2 (22.6, 55.4)	31.7 (21.7, 50.6)	0.12
AST (U/L)	41.25 (27.9, 69.7)	41.6 (28.4, 69.4)	40.2 (27.05, 71.7)	0.647
Total Bilirubin (μmol/L)	19.0 (12.8, 31.6)	18.8 (12.8, 31.85)	19.2 (12.8,30.75)	0.61
γ-GGT (U/L)	57.7 (31.98, 120.78)	57.9 (31.55, 120.5)	56.5 (32.7, 123.05)	0.729
Albumin (g/L)	35.75 ± 6.37	35.39 ± 6.40	35.47 ± 6.31	0.749
LDH (U/L)	177.3 (160.5, 212.53)	178.0 (159.95, 214.65)	176.0 (161.1, 209.75)	0.465
ALP (U/L)	93.0 (72.5, 1301.9)	92.3 (72.5, 130.5)	95.8 (72.65, 133.75)	0.242
Cholinesterase (U/L)	4284 (2705.75, 6200.25)	4280 (2689.5, 6216)	4306 (2761.5, 6179.0)	0.822
Cholesterol (mmol/L)	3.63 (3.13, 4.04)	3.63 (3.11, 4.07)	3.63 (3.15, 4.03)	0.81
Creatinine (µmol/L)	66.0 (57.0, 77.0)	66.0 (57.25, 77.0)	65.0 (56.05, 76.0)	0.072
Prothrombin activity (%)	76.24 ± 18.08	76.26 ± 17.88	76.19 ± 18.52	0.874
INR	1.17 ± 0.23	1.17 ± 0.23	1.17 ± 0.23	0.944
AFP (ng/ml) (<400/≥400)	1996/674 (74.8%/25.2%)	1391/470 (74.7%/25.3%)	605/204 (74.8%/25.2%)	0.983
CRP (mg/L)	7. 0 (1.8, 26.97)	7.3 (2.0, 26.57)	6.6 (1.7, 27.35)	0.762
T cell counts (cells/μL)	734.5 (470.75, 1072.5)	735 (468.0, 1104)	734.0 (474.0, 1027.0)	0.52
CD4 T cell counts (cells/μL)	443.0 (279.0, 653.0)	446.0 (281.0, 664.0)	438.0 (274.0, 622.0)	0.18
CD8 T cell counts (cells/μL)	247.0 (150.0, 402.0)	247.0 (149.0, 405.0)	249.0 (151.0, 395.0)	0.899
CD4/CD8	1.73 (1.26, 2.38)	1.71 (1.19, 2.37)	1.76 (1.27, 2.39)	0.135
Child-Pugh stage (A/B/C)	1385/935/350 (51.9%/35.0%/13.1%)	961/656/244 (51.6%/35.2%/13.1%)	424/279/106 (52.4%/34.5%/13.1%)	0.924
**Tumor-related indicators**				
Tumor multiplicity (solitary/multiple)	1508/1162 (56.5%/43.5%)	1065/796 (57.2%/42.8%)	443/366 (54.8%/45.2%)	0.237
Tumor size, cm (<5/≥ 5)	1785/885 (66.9%/33.1%)	1260/601 (67.7%/32.3%)	525/284 (64.9%/35.1%)	0.156
Portal Vein Tumor Thrombus (Yes/No)	553/2117 (20.7%/79.3%)	379/1482 (20.4%/79.6%)	174/635 (21.5%/78.5%)	0.503
**BCLC staging**				
0-A	982/1688 (36.8%/63.2%)	695/1166 (37.3%/62.7%)	287/522 (35.5%/64.5%)	0.357
B	848/1822 (31.8%/68.2%)	589/1272 (31.6%/68.4%)	259/550 (32.0%/68.0%)	0.852
C	485/2185 (18.2%/81.8%)	331/1530 (17.8%/82.2%)	154/655 (19.0%/81.0%)	0.441
D	355/2315 (13.3%/86.7%)	246/1615 (13.2%/86.8%)	109/700 (13.5%/86.5%)	0.859
**Types of treatment**				
Resection (Yes/No)	226/2444 (8.5%/91.5%)	152/1709 (8.2%/91.8%)	74/735 (9.1%/90.9%)	0.403
Minimally invasive (Yes/No)	1809/861 (67.8%/32.2%)	1275/586 (68.5%/31.5%)	534/275 (66.0%/34.0%)	0.203
Palliative (Yes/No)	635/2035 (23.8%/76.2%)	434/1427 (23.3%/76.7%)	201/608 (24.8%/75.2%)	0.395

SD, standard deviation; NLR, Neutrophil‐lymphocyte ratio; ALT, alanine aminotransferase; AST, aspartate aminotransferase; γ-GGT, γ-glutamyl transferase, LDH, lactate dehydrogenase; ALP, alkaline phosphatase; INR, international normalized ratio; AFP, alpha-fetoprotein; CRP, C reactive protein.

### Overall Survival Analysis

**Figure 1:**
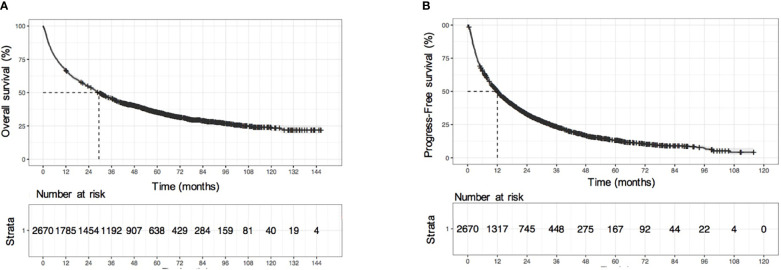
Survival analysis of all HCC patients. **(A)** KM survival curves of overall survival (OS), **(B)** KM survival curves of progression-free survival (PFS).

The median follow-up time was 72.6 months (95% CI: 70.3-76.0). The median OS time was 29.3 (95% CI: 27.3-31.9) months, and the median progression-free survival (PFS) was 12.0 (95% CI: 11.3-13.0) months. The 1-year, 3-year, 5-year, 7-year, and 10-year cumulative OS rates were 66.9%, 45.7%, 34.9%, 28.8%, and 22.6%, respectively (**Figure 1A**), and the cumulative PFS at 1-year, 3-years, 5-years, and 7-years was 50%, 23.3%, 13.1%, and 8.7%, respectively (**Figure 1B**). The cumulative disease-free survival (DFS) at 1-year, 3-years and 5-years was 73.7%,46.9%, and 36.7%, respectively, in the HCC patients with resection and minimally invasive. There was no significant difference between the survival time of training and validation sets [training set: 28.4 (95% CI: 26.1–32.5) months vs. validation set: 30.3 (95% CI: 27.4–35.4) months, P = 0.683].

### Development of ANN Model

**Figure 2:**
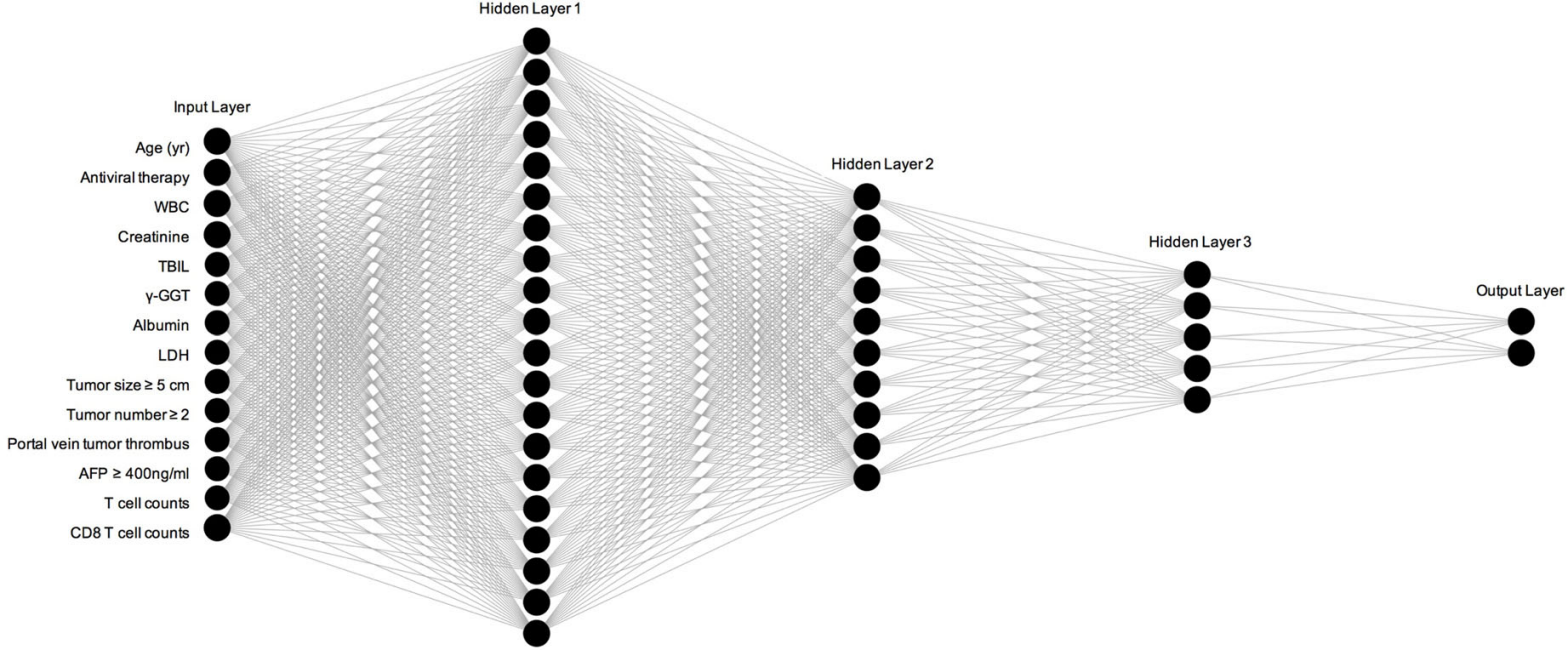
Construction and specific structure of ANN model. WBC, white blood cells; TBIL, total bilirubin; LDH, lactate dehydrogenase; AFP, Alpha fetoprotein.

The results of univariate and multivariate Cox proportional hazard regression analyses are shown in **Table 2**. We identified age at diagnosis, alcohol abuse, tumor size ≥ 5 cm, tumor number ≥ 2, portal vein tumor thrombus (PVTT), Child-Pugh stage C, white blood cells, total bilirubin, lactate dehydrogenase, γ-glutamyl transferase, alkaline phosphatase, creatinine, AFP ≥ 400 ng/ml, and C-reactive protein as independent risk factors for overall survival in HCC patients. In addition, we found antiviral therapy, albumin, T cell count, and CD8 T cell count to be the protective factors. These parameters were included in the ANN model. As shown in **Figure 2**, our ANN model has 14 clinical or biochemical parameters as input neurons and two corresponding clinical outcomes as output neurons. Each neuron is connected by weighted links. To improve the performance of the multilayer perceptron (MLP), after several rounds of debugging and testing, we added three hidden layers.

**Table 2:** Factors associated with overall survival of patients with HCC.

Variables	Univariate analysis			Multivariate analysis		
	HR	95%CI	*P values*	HR	95%CI	*P values*
Age (yr)	1.015	1.010-1.021	<0.0001	1.012	1.006-1.018	<0.0001
Gender (male)	1.250	1.088-1.438	0.002			
Family history of HCC	0.902	0.653-1.246	0.532			
Smoking history	1.211	1.083-1.354	0.001			
Alcohol abuse	1.291	1.154-1.444	<0.0001	1.129	1.005-1.267	0.041
Diabetes	1.011	0.884-1.154	0.878			
Hypertension	0.973	0.858-1.103	0.670			
Hyperlipidemia	0.984	0.788-1.229	0.887			
Coronary artery disease	0.922	0.662-1.285	0.633			
Aetiology			<0.0001			
HBV	REF					
HCV	0.847	0.684-1.049	0.128			
Alcohol abuse	1.307	1.095-1.558	0.003			
Other	1.507	1.151-1.973	0.003			
HBeAg postive	1.011	0.889-1.149	0.872			
HBV-DNA ≥ 500 IU/ml	1.618	1.431-1.828	<0.0001			
Antiviral therapy	0.536	0.456-0.627	<0.0001	0.870	0.767-0.987	0.030
Tumor size ≥5cm	2.658	2.372-2.978	<0.0001	1.746	1.541-1.977	<0.0001
Tumor numbers ≥2	1.793	1.604-2.005	<0.0001	1.299	1.157-1.458	<0.0001
Portal vein tumor thrombus	3.138	2.764-3.564	<0.0001	1.478	1.283-1.704	<0.0001
Child-Pugh stage			<0.0001			0.007
A	REF			REF		
B	2.074	1.835-2.344	<0.0001	1.159	0.997-1.347	0.054
C	3.137	2.673-3.681	<0.0001	1.436	1.146-1.799	0.002
WBC (10^9^/L)	1.041	1.019-1.064	<0.0001	1.054	1.031-1.076	<0.0001
NLR	1.043	1.033-1.054	<0.0001			
Platelets (10^9^/L)	1.000	1.00-1.001	0.288			
Hemoglobin (g/L)	0.990	0.998-0.992	<0.0001			
ALT (U/L)	1.001	1.000-1.002	0.001			
Total Bilirubin (umol/L)	1.004	1.003-1.005	<0.0001	1.002	1.001-1.003	<0.0001
Albumin (g/L)	0.944	0.936-0.952	<0.0001	0.981	0.969-0.993	0.002
LDH (U/L)	1.001	1.001-1.001	<0.0001	1.001	1-1.001	<0.0001
γ-GGT (U/L)	1.002	1.002-1.002	<0.0001	1.001	1-1.001	<0.0001
ALP (U/L)	1.003	1.003-1.003	<0.0001	1.001	1.001-1.002	<0.0001
Creatinine (µmol/L)	1.002	1.002-1.003	<0.0001	1.002	1.001-1.002	<0.0001
Prothrombin activity (%)	0.983	0.98-0.986	<0.0001			
AFP ≥ 400 ng/ml	2.196	1.945-2.479	<0.0001	1.684	1.478-1.918	<0.0001
CRP (mg/L)	1.009	1.008-1.011	<0.0001	1.004	1.002-1.006	<0.0001
T cell counts (/μL)	0.999	0.999-0.999	<0.0001	0.999	0.999-1	<0.0001
CD8 T cell counts (/μL)	0.998	0.997-0.998	<0.0001	0.999	0.998-0.999	<0.0001
CD4 T cell counts (/μL)	0.998	0.998-0.999	<0.0001			

WBC, white blood cells; NLR, Neutrophil‐lymphocyte ratio; ALT, alanine aminotransferase; LDH, lactate dehydrogenase; γ-GGT, γ-glutamyl transferase; ALP, alkaline phosphatase; AFP, alpha-fetoprotein; CRP, C-reactive Protein.

### Performance Comparison of ANN Model With Other Models

**Figure 3:**
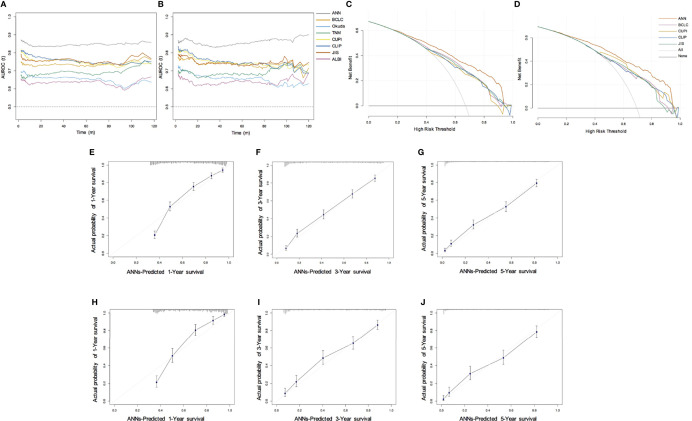
Performance of ANN model. **(A, B)** Time-dependent area under the ROC curve for the long-term risk prediction of HCC patients in the training **(A)** and validation cohort **(B)**; **(C, D)** Decision curve analysis for predicting the OS in the training **(C)** and validation cohort **(D)**; **(E–G)** Calibration curve for predicting 1-year, 3-year, and 5-year OS in the training cohort; **(H–J)** Calibration curve for predicting 1-year, 3-year, and 5-year OS in the validation cohort.

In the training set, the AUC values of 1-year, 3-year and 5-year survival predicted by the ANN model were 0.838, 0.833, and 0.843, respectively, and the C index was 0.769; the AUC values of 1-year, 3-year and 5-year survival by the model constructed by traditional Cox regression analysis were 0.736, 0.701, and 0.685, respectively, and the C index was 0.712, which was significantly lower than that of the ANN model (P < 0.05) (**Table 3**). The results indicate that the ability of the ANN model to distinguish the survival outcome of liver cancer patients was significantly higher than that of the traditional Cox regression model. Similar results were obtained for the validation set. The AUC value of the ANN was significantly higher than that of the Cox model but there was no difference in the C index between the two models. Furthermore, we compared the ANN model with other classical models for prognosis evaluation of HCC, such as the BCLC, TNM, Okuda, CUPI, CLIP, JIS, and ALBI models, and found that the AUC value and C index of the ANN model in the prediction of OS and DFS outperformed them in both the training and validation sets (**Table 4**, **Table S1**). Considering the continuity of survival time of liver cancer, we found that the time-dependent AUC values of the ANN model were all higher than those of the other models in the training and validation sets, as expected (**Figures 3A, B**).

**Table 3:** Comparison of the performance and discriminative ability between ANNs and COX model.

Corhort	Models	1-yr AUROC (95%CI)	3-yr AUROC (95%CI)	5-yr AUROC (95%CI)	LR χ^2^	C-index (95%CI)	AIC
Training*	ANNs	0.838 (0.819-0.857)	0.833 (0.815-0.851)	0.843 (0.825-0.861)	955.800	0.769 (0.757-0.782)	16502.210
	COX model	0.736 (0.711-0.761)	0.701 (0.677-0.725)	0.685 (0.657-0.713)	627.900	0.712 (0.696-0.728)	16850.130
Validation**	ANNs	0.871 (0.845-0.897)	0.831 (0.804-0.859)	0.848 (0.821-0.874)	448.000	0.773 (0.755-0.792)	6425.971
	COX model	0.754 (0.718-0.79)	0.7 (0.664-0.736)	0.722 (0.682-0.763)	516.200	0.778 (0.76-0.797)	6383.769

*In training cohort, p values for LR Chi-square test and C-index comparison between ANN model and COX model were both <0.001.

**In validation cohort, p values for LR Chi-square test and C-index comparison between ANN model and COX model were 0.001 and 0.718.

**Table 4:** Comparison of the performance and discriminative ability between the ANNs model and conventional models.

Corhort	Models	1-yr AUROC (95%CI)	3-yr AUROC (95%CI)	5-yr AUROC (95%CI)	C-index (95%CI)
Training	ANNs	0.838 (0.819-0.857)	0.833 (0.815-0.851)	0.843 (0.825-0.861)	0.769 (0.757-0.782)
	BCLC	0.731 (0.707-0.755)	0.731 (0.708-0.754)	0.735 (0.712-0.758)	0.674 (0.66-0.688)
	TNM	0.674 (0.649-0.7)	0.689 (0.665-0.713)	0.692 (0.667-0.716)	0.633 (0.618-0.648)
	Okuda	0.67 (0.644-0.696)	0.661 (0.636-0.685)	0.675 (0.65-0.7)	0.626 (0.612-0.64)
	CUPI	0.764 (0.741-0.787)	0.759 (0.738-0.781)	0.757 (0.735-0.779)	0.701 (0.687-0.716)
	CLIP	0.788 (0.766-0.81)	0.759 (0.737-0.781)	0.755 (0.733-0.777)	0.707 (0.693-0.721)
	JIS	0.759 (0.737-0.781)	0.757 (0.736-0.779)	0.764 (0.741-0.786)	0.697 (0.683-0.711)
	ALBI	0.63 (0.603-0.656)	0.639 (0.614-0.664)	0.654 (0.628-0.68)	0.604 (0.59-0.618)
					
Validation	ANNs	0.871 (0.845-0.897)	0.831 (0.804-0.859)	0.848 (0.821-0.874)	0.773 (0.755-0.792)
	BCLC	0.767 (0.734-0.801)	0.739 (0.704-0.773)	0.748 (0.713-0.783)	0.684 (0.665-0.704)
	TNM	0.694 (0.656-0.732)	0.68 (0.643-0.717)	0.689 (0.651-0.727)	0.633 (0.612-0.654)
	Okuda	0.688 (0.648-0.727)	0.657 (0.619-0.694)	0.666 (0.628-0.704)	0.631 (0.61-0.652)
	CUPI	0.791 (0.758-0.824)	0.749 (0.715-0.782)	0.751 (0.717-0.785)	0.705 (0.683-0.726)
	CLIP	0.802 (0.769-0.835)	0.749 (0.715-0.782)	0.743 (0.709-0.777)	0.706 (0.685-0.726)
	JIS	0.772 (0.739-0.805)	0.739 (0.704-0.774)	0.738 (0.702-0.774)	0.691 (0.67-0.712)
	ALBI	0.641 (0.6-0.681)	0.62 (0.582-0.658)	0.644 (0.605-0.683)	0.604 (0.582-0.625)

Considering that different etiologies, liver functions, and treatment methods may affect the prognosis of HCC patients, we further analyzed the performance of these subgroups. In terms of age, sex, etiology, AFP level, Child-Pugh grade, and era of diagnosis and treatments, we also compared the AUC value, C index of 1-year, 3-year, 5-year survival, and DFS and found that the ANN model was higher than other models (**Table S2**, **Table S3**, **Table S4**).

By drawing the calibration curve, we showed that the ANN model can predict the 1-year, 3-year, 5-year OS probabilities of HCC patients and the corresponding actual observation probabilities (**Figures 3E–J**). In the training and validation sets, the ANN model had a good fit slope in predicting 1-year, 3-year, 5-year OS. In addition, compared with the BCLC, TNM, Okuda, CUPI, CLIP, JIS, and ALBI models, our model showed significant net clinical benefits and improved the overall survival of HCC patients in DCA (**Figures 3C, D**). These results show that the ANN model has a better clinical practicability than other models.

### Application of ANN Model for Risk Stratification

**Figure 4:**
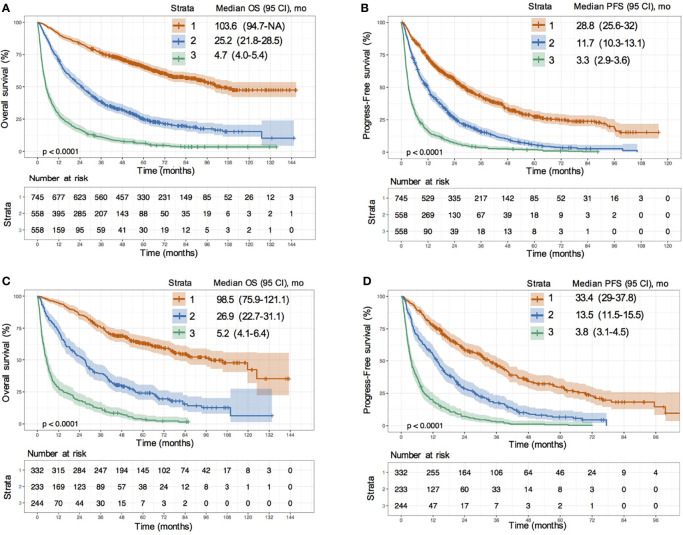
Risk stratification survival analysis of HCC patients divided by ANN model. **(A, B)** KM survival curves of overall survival (OS) **(A)** and progression-free survival (PFS) **(B)** in training cohort; **(C, D)** KM survival curves of OS **(C)** and PFS **(D)** in validation cohort.

According to the 40% and 70% digits of the ANN model score, all patients were divided into three levels: low risk (stratum 1), medium risk (stratum 2) and high risk (stratum 3). In the training set, compared with the low-risk group, the hazard ratio (HR) values of OS for medium-risk and high-risk groups were 3.01 (95% CI: 2.59–3.50; P < 0.0001) and 8.11 (95% CI: 7.0–9.4; P < 0.0001), respectively (**Figure 4A**); the HR values of PFS were 2.15 (95% CI: 1.90–2.45; P < 0.0001) and 4.98 (95% CI: 4.38–5.66; P < 0.0001), respectively (**Figure 4B**). In the validation set, compared with the low-risk group, the HR values of OS for medium risk and high-risk groups were 3.12 (95% CI: 2.50–3.89; P < 0.0001) and 8.65 (95% CI: 6.93–10.79; P < 0.0001), respectively (**Figure 4C**); the HR values of PFS were 2.28 (95% CI: 1.87–2.77; P < 0.0001) and 5.58(95% CI: 4.59–6.80; P <0.0001), respectively (**Figure 4D**). Whether in the training or validation set, the ANN model could effectively distinguish all patients according to their different death risks.

We further drew Kaplan-Meier (KM) survival curves of the ANN model after risk stratification in the different etiology, liver function, inclusion time and treatment methods subgroups (**Figures S1**, **S2**). There was no difference between the medium- and low-risk patients (log-rank P value= 0.06) (**Figure S1G**) in Child-Pugh C (CTP C) grade. In the remaining sublayers, the ANN model could distinguish the patients well. The median survival time and HR values of OS in the different risk groups for all sublayers are shown in **Table S5**. The same results were obtained in the survival curves of the ANN model after risk stratification for the DFS (**Figure S3**) and early recurrence (**Figure S4** and **Table S6**).

### Prognostic Value of T Cell and CD8T Cell Counts in HCC Patients

**Figure 5:**
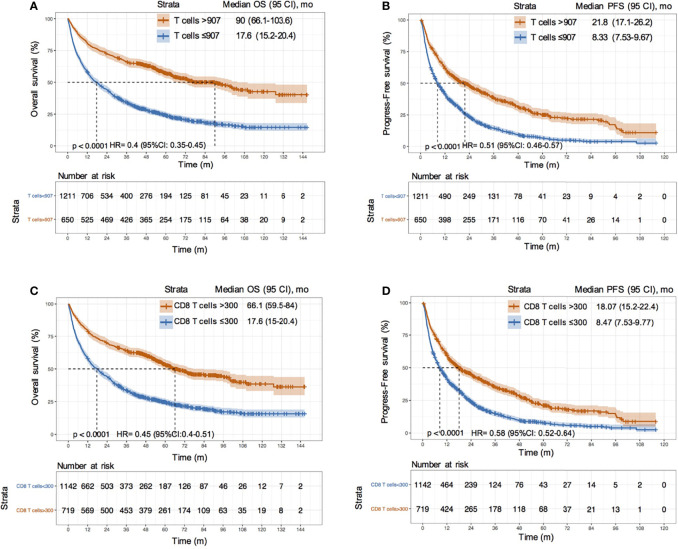
Survival analysis between HCC patients with different T cell and CD8 T cell counts. **(A, B)** KM curves of OS and PFS between HCC patients with T cells > 907/μL and ≤ 907/μL in training cohort; **(C, D)** KM curves of OS and PFS between HCC patients with CD8 T cells > 300/μL and ≤ 300/μL in training cohort.

We used 907 cells/μL as the cutoff value of T cell counts and 300 cells/μL as the cutoff value of CD8 T cell counts according to the maximum value of the Youden index. We divided all patients into two groups based on the cutoff values and assessed the overall survival, as visualized by the Kaplan–Meier survival curves. The median survival time of patients with T cell counts > 907 cells/μL was more than five times longer than that of patients with T cell counts ≤ 907 cells/μL (90 vs. 17.6 months) in the training set. The risks of death and progression in patients with a high frequency of T cells were significantly reduced (death risk: HR = 0.4, 95% CI: 0.35–0.45; progression risk: HR = 0.51, 95% CI: 0.48–0.57; P < 0.0001) (**Figures 5A, B**). The same results were obtained after a grouping based on the cut-off value of CD8 T cells (**Figures 5C, D**). We also estimated the discrimination and prognostic values of circulating T cells and CD8 T cells in different etiologies and treatment sublayers (**Figures S5**, **S6**). The results suggested that an increase in T cell counts and CD8 T cell counts in HCC patients could improve the survival rate and prolong the survival time, especially in patients who underwent resection (HR value < 0.35, P < 0.001).

## Discussion

Recently, machine learning has been successful in cancer detection, prognostic risk stratification, and clinical decision-making for breast, prostate, lung, and other cancers (22, 23, 26, 27). Although artificial intelligence has been applied in various imaging diagnoses and prognosis evaluations after different therapies of HCC patients, it is rarely applied to the OS of HCC patients (28). In this study, a machine learning method was used to build an ANN prediction model suitable for individual applications, which can calculate the death probability of HCC patients. This model is a simple and easy-to-use calculator, integrating tumor characteristics of HCC patients: tumor size, number, portal vein tumor thrombus, AFP, liver function, albumin, total bilirubin, γ-GGT, LDH, inflammatory index, white blood cell counts, antiviral therapy, and immune index—T cell counts and CD8 T cell counts. The C index of the prediction model in this study is greater than 0.75 in the training and validation sets and the AUC value is greater than 0.8, which indicates the ANN model is more reliable.

Machine learning is the most common approach to artificial intelligence and can mimic human cognitive functions through machines or algorithms. ANNs can build probabilistic or statistical models and maximize the accuracy of predictions. ANNs are able to learn and repeatedly train clinical data by imitating the information processing function of human brain synapses, thereby acquiring decision-making ability and simple judgment ability similar to that of humans (29). Compared with conventional Cox or logistic regression analyses, ANNs have the advantages of nonlinear mapping and high accuracy. ANNs can adjust the weights between input and output values and minimize the error between actual and expected outputs. In this study, the AUC values of the ANN model were significantly higher than those of the Cox model for predicting the short- and long-term survival of HCC patients. Moreover, the time-dependent ROC curve also revealed that the ANN model outperformed other scoring systems, including BCLC, TNM, JIS, CLIP, CUPI, Okuda, and ALBI, in predicting HCC outcomes under any survival time. Similar to this study, we have used ANNs to develop a model with good accuracy to predict the progression-free survival of HBV-HCC patients (26). The AUC value and C-index were 0.866 and 0.782, respectively, which were superior to the above scoring systems. The ANN system could help doctors and patients make better clinical decisions, screen timely, and slow the progression of the disease.

Therapies play a decisive role in the prognosis of HCC patients. Several studies have focused on a machine learning approach for predicting the response and prognosis of different treatments (21, 30, 31). Liu et al. applied random forest feature selection, a support vector machine (SVM), and multitask deep learning to build a survival-sensitive risk stratification model in 243 HCC patients receiving TACE (30). Saillard et al. used deep learning algorithms to construct a model for predicting survival by analyzing whole-slide digitized histological slides from 194 HCC patients after resection (21). At present, most studies are based on tumor histopathology and radiomics-based features to construct survival prediction models for patients after certain treatments. However, a majority of HCC patients cannot obtain tumor histopathological sections because once discovered, the patients are in the middle and advanced stages and have no chance of surgery. In this study, only 8.5% of the patients underwent resection. Imaging features have a significant heterogeneity among different equipment, parameter settings, and researchers’ extraction methods. Therefore, the ANN model using clinical and laboratory characteristics is not only noninvasive but also convenient and accurate. We also verified the predictive efficacy of the proposed model in different treatment subgroups and found that the AUC values for predicting 1-, 3-, and 5-year survival were all higher than those of other scoring systems in the resection, minimally invasive, and palliative groups. Moreover, the ANN model had a good discriminatory power in different treatment subgroups.

The immune system is an important way to exert antitumor effects. Several studies have shown that a high density of tumor-infiltrating lymphocytes is correlated with good clinical outcomes in different types of tumors (17–19). Unitt reported that a decrease in tumor-infiltrating lymphocytes (TILs) is an independent risk factor for HCC recurrence after liver transplantation (32). In addition, previous studies also found that a high density of CD3 and CD8 T cell infiltration in the tumor area can significantly reduce the recurrence rate of HCC patients after resection and improve overall survival (19). However, because of the limitations in tumor tissue acquisition, the relationship between immune cells and prognosis in patients with intermediate and advanced HCC who cannot undergo surgical resection remains unclear. Through this large cohort study, we found that increased circulating T cell and CD8 T cell counts could improve the survival rate and prolong survival time. This is consistent with the results for lung, colorectal, and other cancers (33–35).

The immune system is a double-edged sword in the development and progression of tumors. A healthy immune system can eliminate tumors by recognizing immune antigens. With the proposal of the tumor immune editing concept, a large number of studies have found that the tumor microenvironment may escape immune elimination by reducing antigenicity and immunogenicity, secreting inhibitory molecules such as tumor growth factor (TGF)-β and interleukin-10, and increasing the proportion of suppressor cell such as regulatory T cells and myeloid-derived suppressor cells (36). T cell exhaustion has become a new focus in tumor immunosuppression in recent years (37). The depletion of T cells cannot effectively recognize tumor antigens and conversely, exhausted T cells with high expression of inhibitory molecules such as PD-1, TIGIT, and TIM-3 gradually lose their proliferation and cytotoxic capacity and further promote tumor progression. Our previous study also found that a high expression of PD-1 and TIGIT on the surface of T cells in HCC patients was associated with disease progression (38). This may explain why the reduced T-cell count in this study was associated with poor outcomes.

Our study has several limitations. First, an ANN with a large number of parameters may be over-fitted or only fit the training data and may not be generalized to other HCC patients. However, the large sample size of this study and fine-tuning of the hyperparameter sets can reduce the effects of overfitting to a certain extent. The ANN model exhibits excellent discrimination and good accuracy in the holdout validation set and several different subgroups, outperforming the routinely used predictive systems. Second, this is a single-center study and most HCC patients have HBV infection. The ANN model should be validated in HCC patients with HCV, alcohol, or nonalcoholic fatty liver disease settings to determine its generalizability.

## Conclusion

In conclusion, this study used artificial neural network to develop a prognostic model to predict long-term overall survival. The ANN model has the advantages of convenience, accuracy, and noninvasiveness. This study identified high frequencies of circulating T cells and CD8 T cells as protective factors. Regular surveillance based on the ANN model indicators may help doctors take clinical decisions and prolong the survival time of HCC patients.

## Data Availability Statement

All datasets generated for this study are included in the article/**Supplementary Material**, further inquiries can be directed to the corresponding author/s.

## Ethics Statement

The studies involving human participants were reviewed and approved by The Ethics Committee of Beijing Ditan Hospital, Capital Medical University. Written informed consent for participation was not required for this study in accordance with the national legislation and the institutional requirements.

## Author Contributions

ZY and JH designed the research. XL and LY assisted with statistical analysis. LY, YH, and XHW were responsible for the patients’ inclusion and follow-up. XL and XHW wrote the manuscript. XBW, YJ, and ZY participated in the revision of the manuscript. All authors contributed to the article and approved the submitted version.

## Funding

This work was supported by the Special Fund of Capital Health Research and Development (No. 2020-2-2173), the National Science Foundation of China (No. 81874435, No. 82104781), Dengfeng Talent Support Program of Beijing Municipal Administration of Hospitals (No. DFL20191803), Fund for Beijing Science & Technology Development of TCM (No. JJ-2020-52), Beijing Hospitals Authority Clinical Medicine Development of Special Funding Support (No. ZYLX202127), Fund of Beijing Ditan Hospital, Capital Medical University (DTYM-202113).

## Conflict of Interest

The authors declare that the research was conducted in the absence of any commercial or financial relationships that could be construed as a potential conflict of interest.

## Publisher’s Note

All claims expressed in this article are solely those of the authors and do not necessarily represent those of their affiliated organizations, or those of the publisher, the editors and the reviewers. Any product that may be evaluated in this article, or claim that may be made by its manufacturer, is not guaranteed or endorsed by the publisher.

## Supplementary Material

Supplementary Figure 1 KM survival curves of overall survival based on ANN model risk stratification in HCC patients with different clinical subgroups. **(A, B)** Patients with different etiology; **(C, D)** Patients with different AFP levels; **(E–J)** Patients with different Child-Pugh stage; **(H, I)** Patients with different inclusion time.

Supplementary Figure 2 Survival analysis based on ANN model risk stratification in HCC patients with different treatments.

Supplementary Figure 3 KM survival curves of disease-free survival based on ANN model risk stratification in HCC patients with training **(A)** and validation group **(B)**.

Supplementary Figure 4 Recurrence analysis based on ANN model risk stratification in HCC patients with different treatments.

Supplementary Figure 5 Survival analysis between HCC patients with different T cells in different etiology **(A, B)**, inclusion time **(C, D)** and treatments groups **(E–G)**.

Supplementary Figure 6 Survival analysis between HCC patients with different CD8 T cells in different etiology **(A, B)**, inclusion time **(C, D)** and treatments groups **(E–G)**.
