# Supplementary figures and images for: A Novel Prognostic Score Based on Artificial Intelligence in Hepatocellular Carcinoma: A Long-Term Follow-Up Analysis
Source: Front Oncol. 2022 May 31;12:817853. doi: 10.3389/fonc.2022.817853 (PMC9195097; doi:10.3389/fonc.2022.817853)

Fig. S1

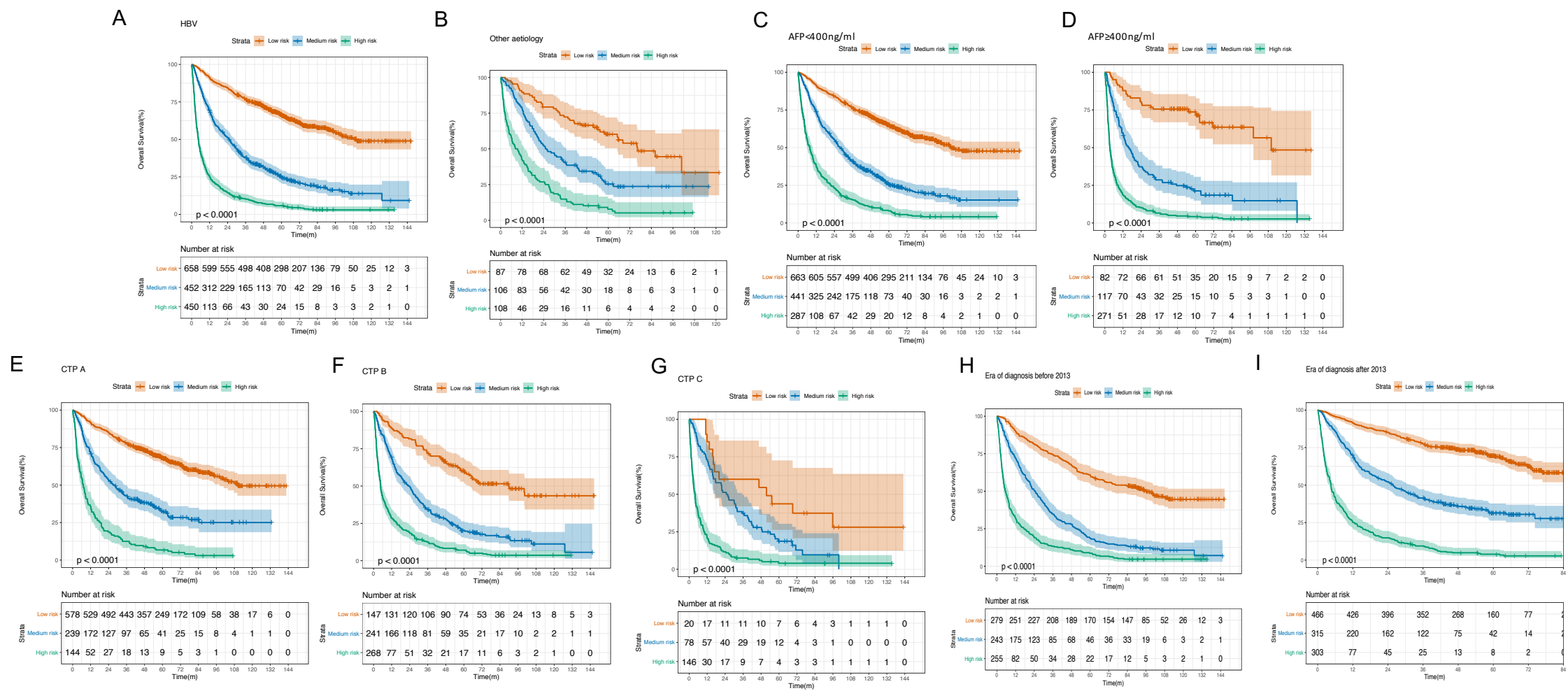

Fig. S2

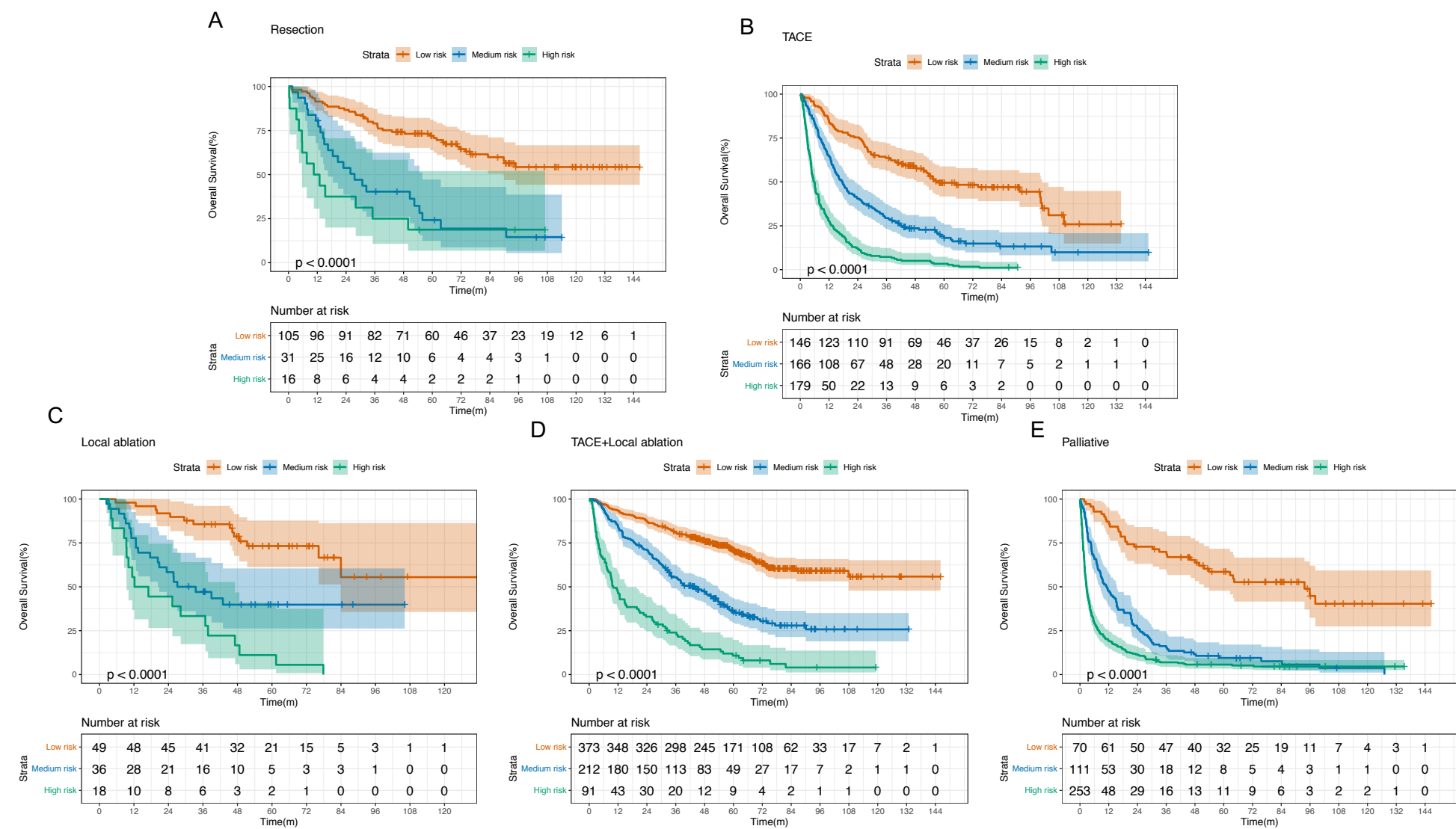

Fig. S3

A

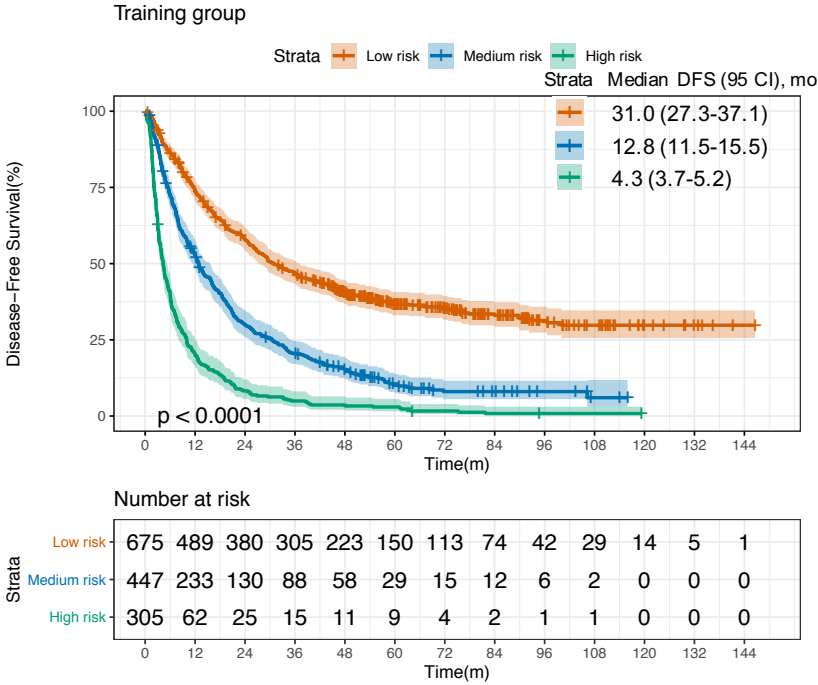

B

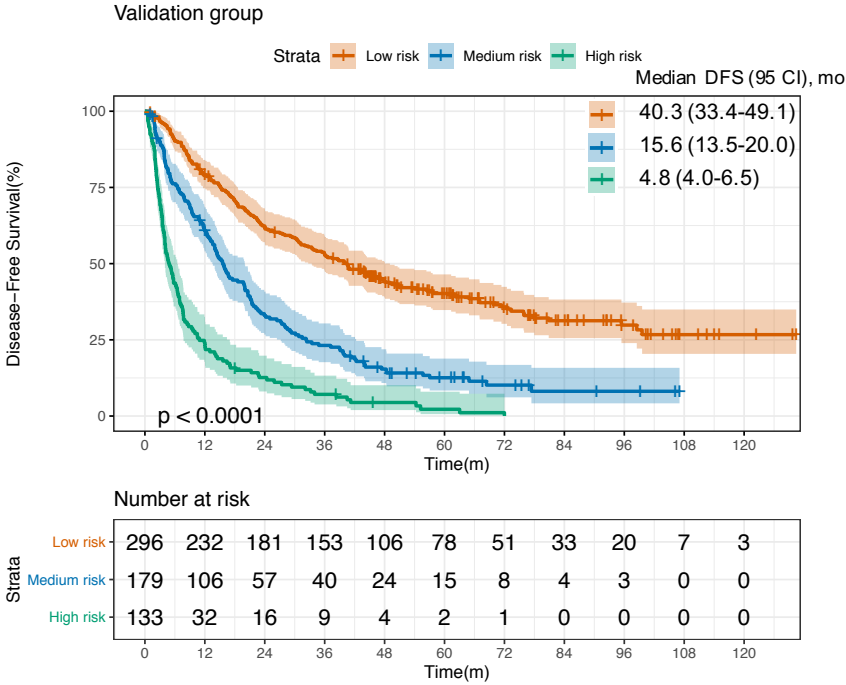

Fig. S4

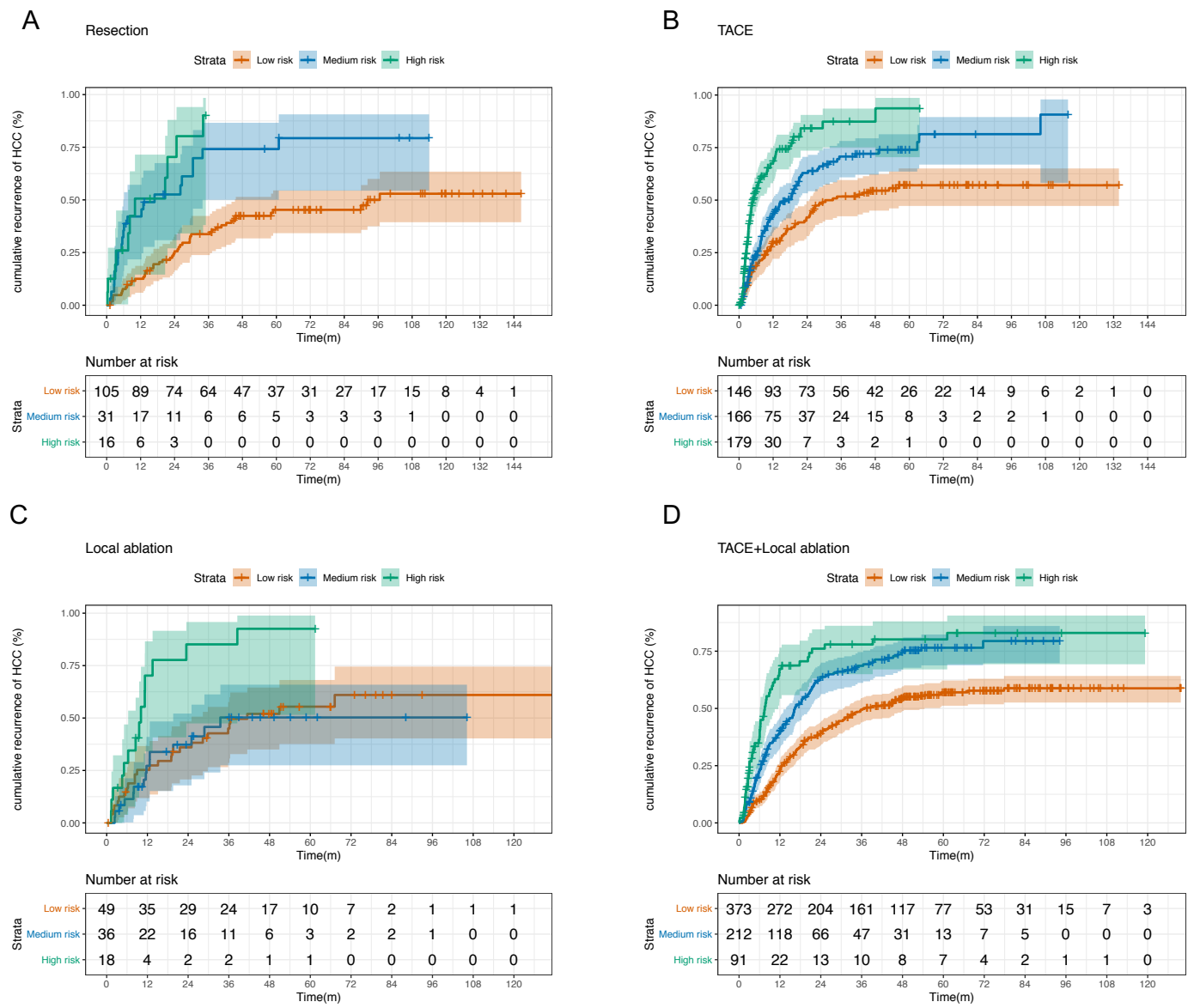

Fig. S5

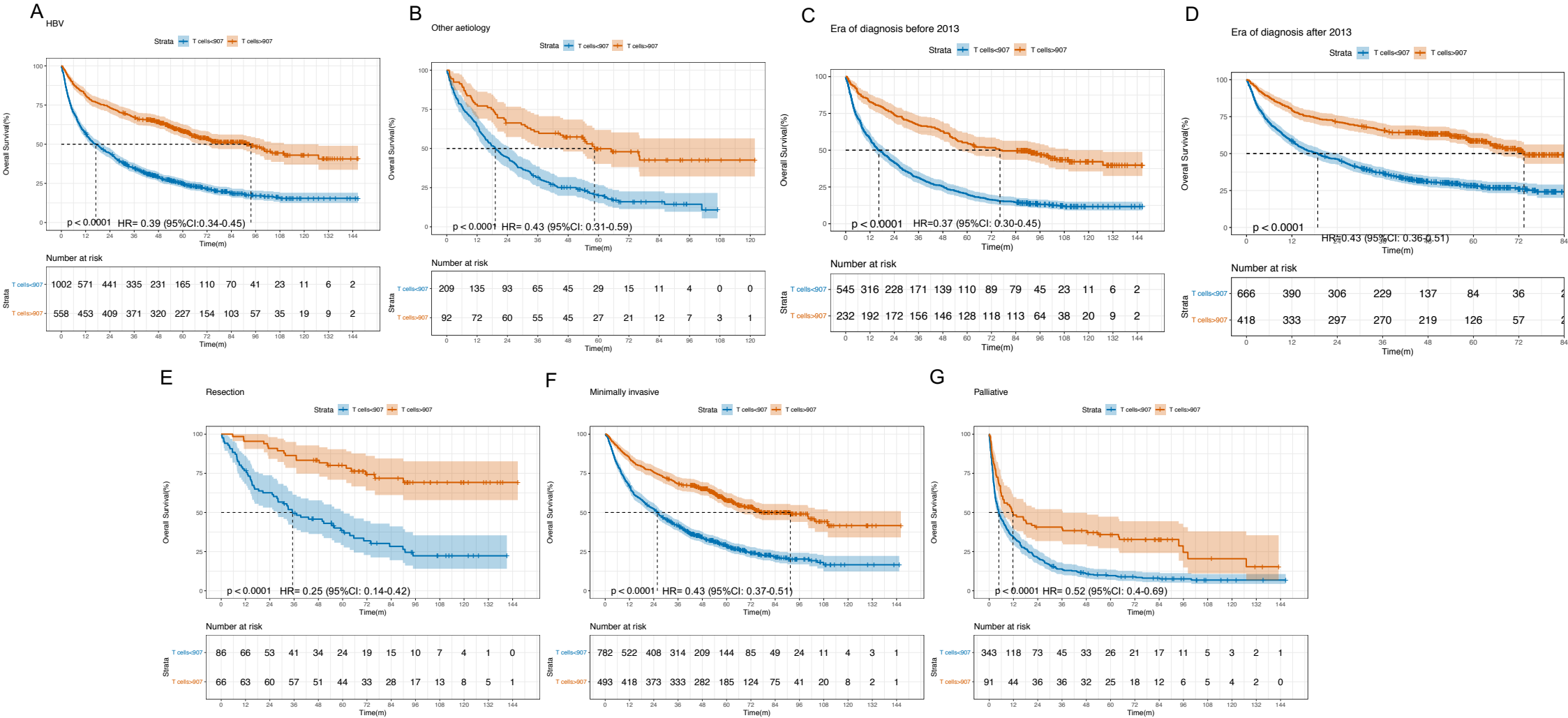

Fig. S6

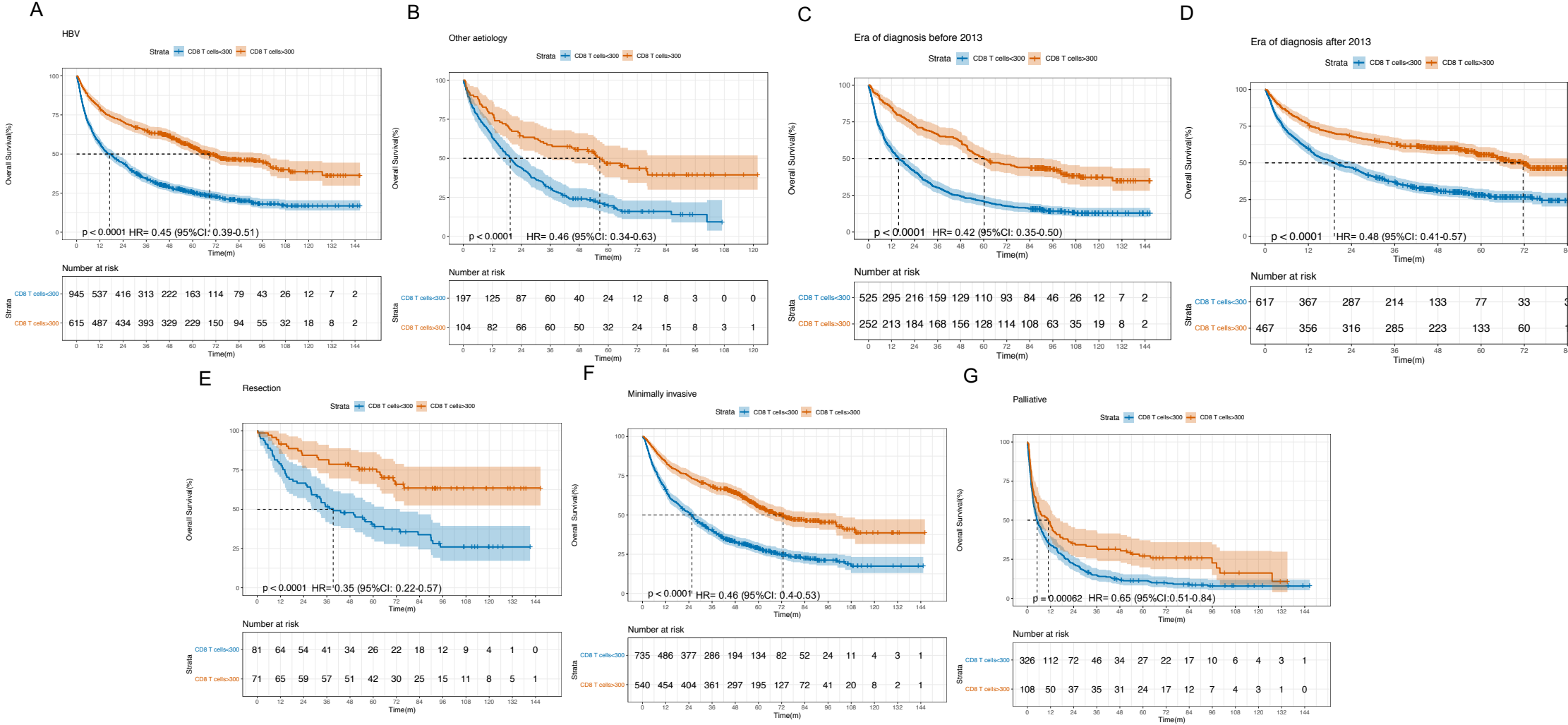

Supplement: Supplementary Figure 1 — KM survival curves of overall survival based on ANN model risk stratification in HCC patients with different clinical subgroups. (A, B) Patients with different etiology; (C, D) Patients with different AFP levels; (E–J) Patients with different Child-Pugh stage; (H, I) Patients with different inclusion time. [file DataSheet_1.pdf]
