# Supplementary material for: A Novel Prognostic Score Based on Artificial Intelligence in Hepatocellular Carcinoma: A Long-Term Follow-Up Analysis
Source: Front Oncol. 2022 May 31;12:817853. doi: 10.3389/fonc.2022.817853 (PMC9195097; doi:10.3389/fonc.2022.817853)
Supplement: Supplementary file 2 [file Table_1.docx]

TableS1 Comparison of the AUROC values of the disease-free survival between the ANNs model and conventional models across different subgroups

TableS2 Comparison of the AUROC among the ANNs model and conventional models across different subgroups

TableS3 Comparison of the C-index among the ANNs model and conventional models across different subgroups

TableS4 Comparison of the performance and discriminative ability of the disease-free survival between the ANNs model and conventional models

TableS5 Survival analyses for the ANNs model across the HCC patients in different subgroups.

TableS6 Recurrence analyses for the ANNs model across the HCC patients in different subgroups.

| Subgroup | n | HR （95%CI） | P-value for HR |
| --- | --- | --- | --- |
| Resction | 152 |  |  |
| Stratum 1 |  | Ref |  |
| Stratum 2 |  | 2.73 (1.64-4.55) | <0.0001 |
| Stratum 3 |  | 4.15 (2.1-8.2) | <0.0001 |
| TACE | 491 |  |  |
| Stratum 1 |  | Ref |  |
| Stratum 2 |  | 1.67 (1.23-2.27) | 0.001 |
| Stratum 3 |  | 3.55 (2.6-4.85) | <0.0001 |
| Local ablation | 103 |  |  |
| Stratum 1 |  | Ref |  |
| Stratum 2 |  | 0.93 (0.49-1.76) | 0.824 |
| Stratum 3 |  | 3.04 (1.55-5.97) | 0.001 |
| TACE+Local ablation | 676 |  |  |
| Stratum 1 |  | Ref |  |
| Stratum 2 |  | 1.81 (1.46-2.25) | <0.0001 |
| Stratum 3 |  | 2.96 (2.21-3.98) | <0.0001 |
